# Supplementary figures and images for: Single-cell transcriptomic profiling reveals a novel signature of necrotizing granulomatous lesions in the lungs of Mycobacterium tuberculosis-infected C3HeB/FeJ mice
Source: Front Immunol. 2025 Aug 6;16:1624072. doi: 10.3389/fimmu.2025.1624072 (PMC12364640; doi:10.3389/fimmu.2025.1624072)

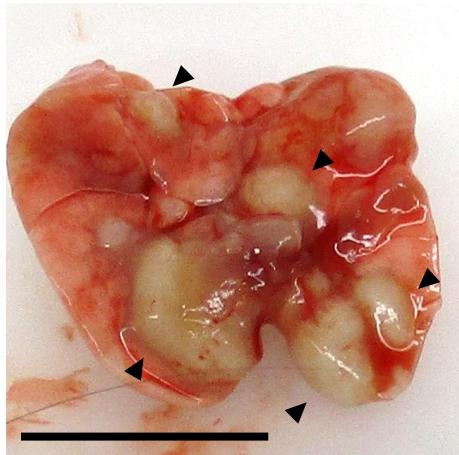

A

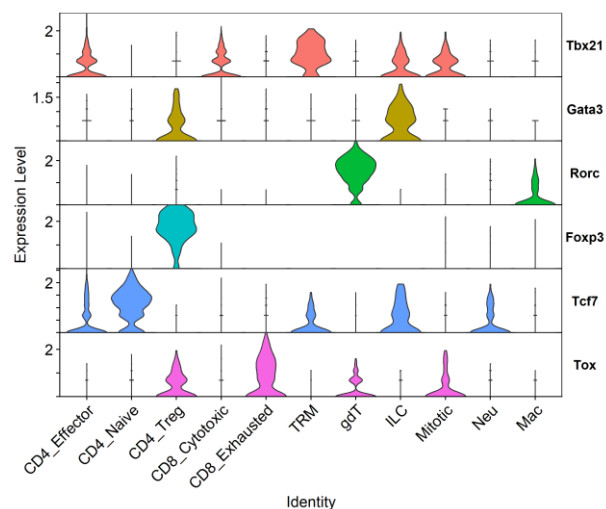

B

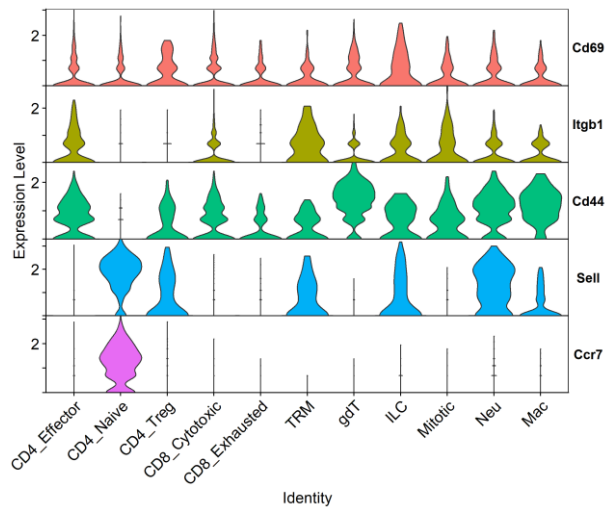

C

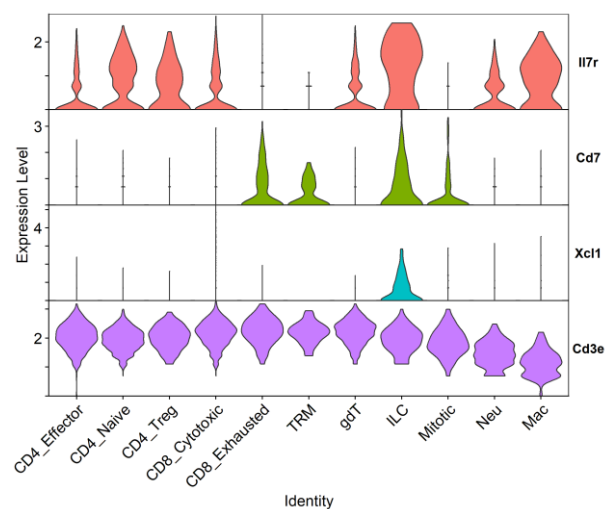

**A Cxcl10**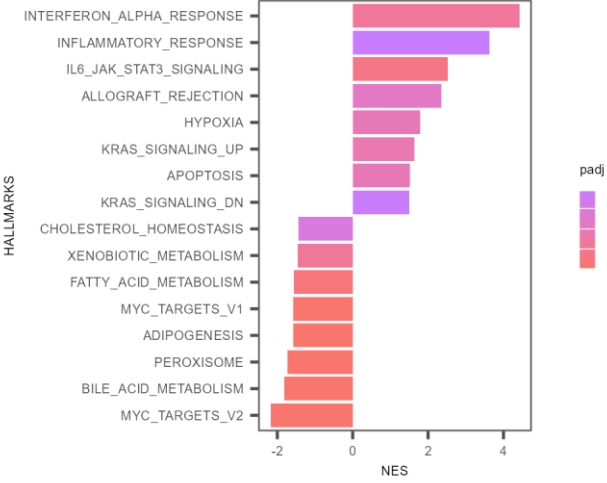**B Clec4e**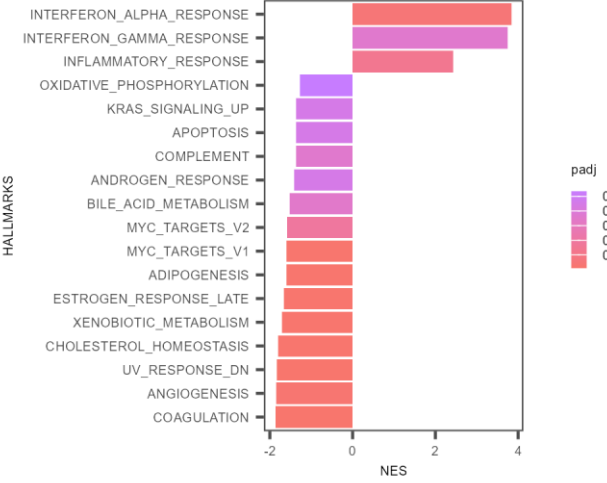**C C1qc**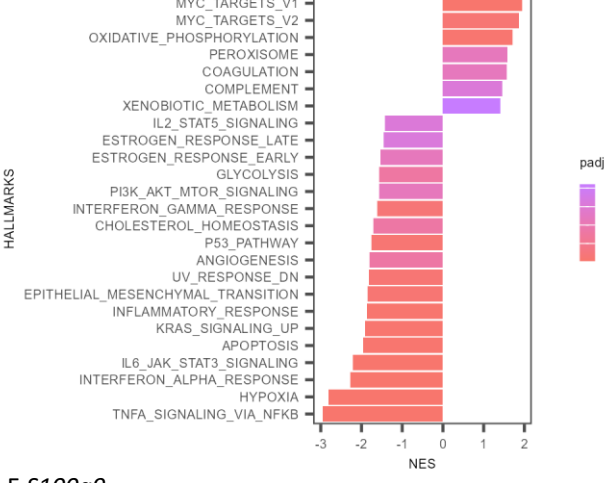**D Ighm**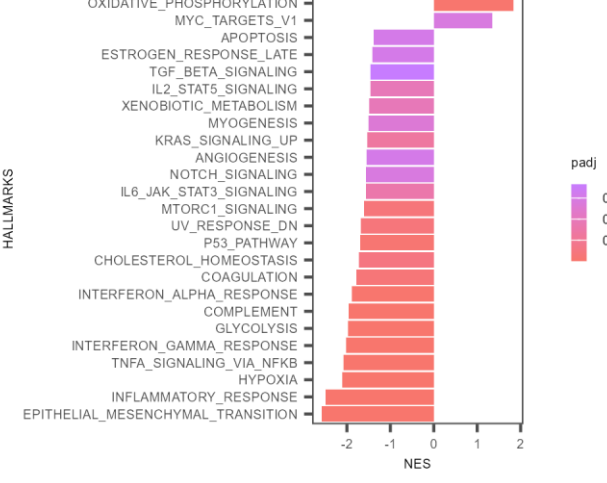**E S100a9**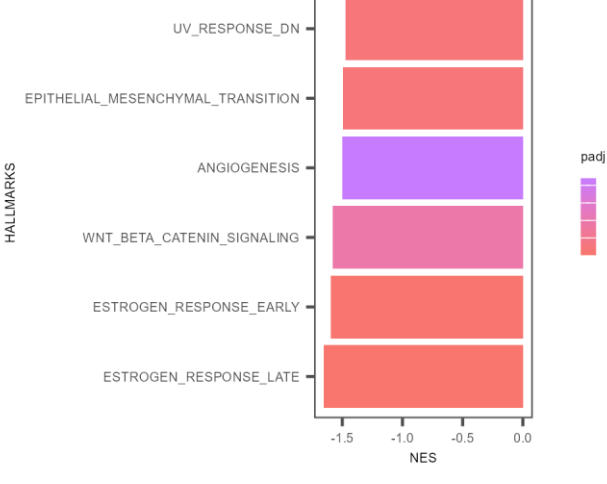**F Siglecf**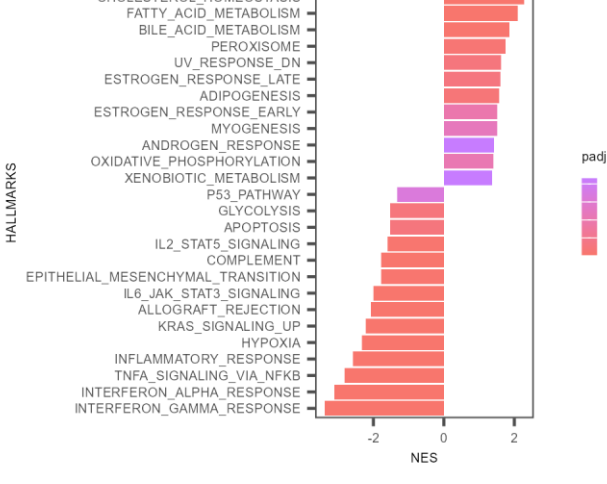

**A**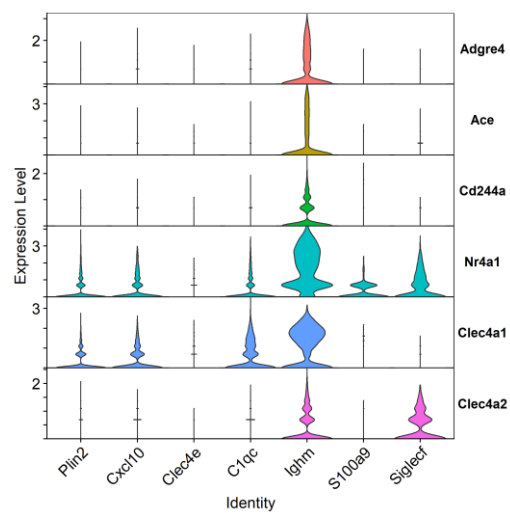**B**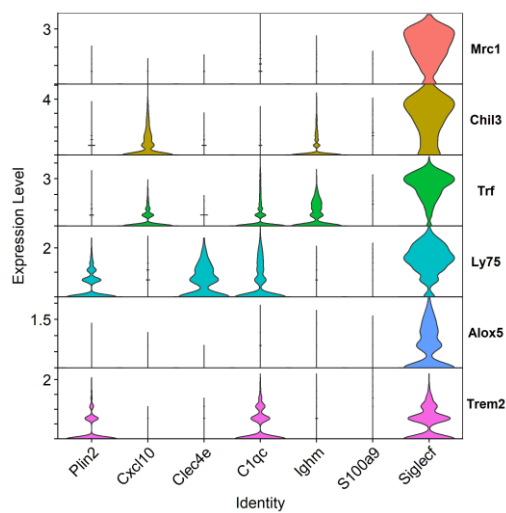**C**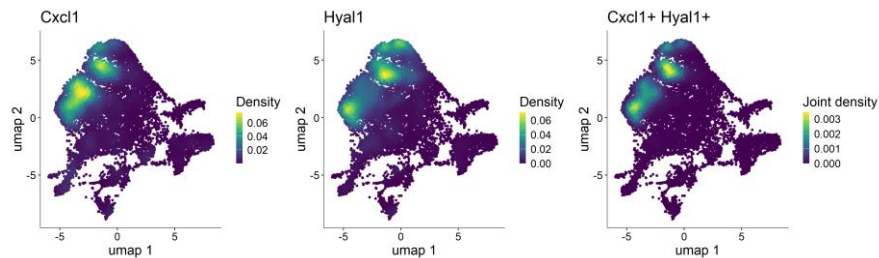

A

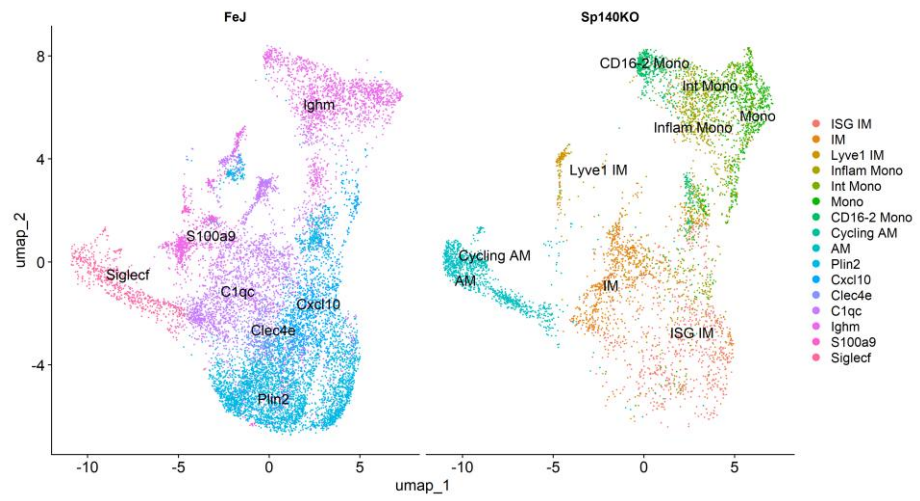

B

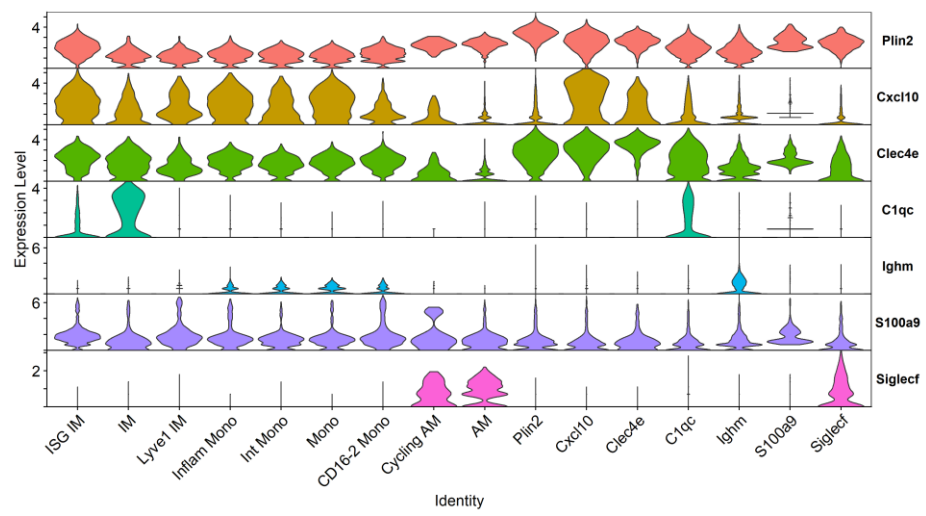

C

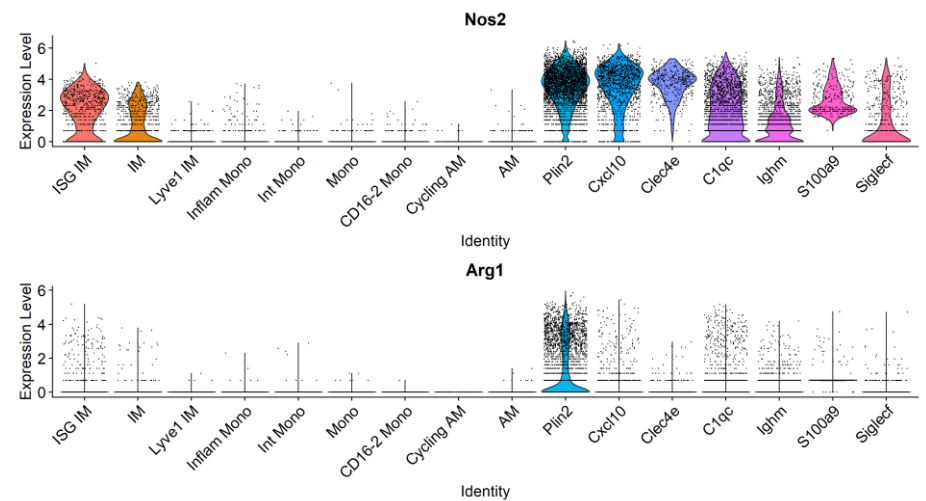

Supplement: Supplementary Figure 1 — Macroscopic image of C3HeB/FeJ mouse lung with necrotizing granulomatous lesions at 12 weeks postinfection (p.i.). Arrowheads indicate necrotizing granulomas. Scale bar, 1 cm. [file Image1.pdf]
